# The Tension at the Top of the Animal Pole Decreases during Meiotic Cell Division

**DOI:** 10.1371/journal.pone.0079389

**Published:** 2013-11-18

**Authors:** Setsuko K. Satoh, Akifumi Tsuchi, Ryohei Satoh, Hiromi Miyoshi, Miyako S. Hamaguchi, Yukihisa Hamaguchi

**Affiliations:** 1 Department of Bioengineering, Graduate School of Bioscience and Biotechnology, Tokyo Institute of Technology, Meguro, Tokyo, Japan; 2 Faculty of Economics and management, Surugadai University, Hanno, Saitama, Japan; 3 Department of Physiology, Kitasato University School of Medicine, Sagamihara, Kanagawa, Japan; 4 Ultrahigh Precision Optics Technology Team, RIKEN Center for Advanced Photonics, Wako, Saitama, Japan; Vanderbilt University Medical Center, United States of America

## Abstract

Meiotic maturation is essential for the reproduction procedure of many animals. During this process an oocyte produces a large egg cell and tiny polar bodies by highly asymmetric division. In this study, to fully understand the sophisticated spatiotemporal regulation of accurate oocyte meiotic division, we focused on the global and local changes in the tension at the surface of the starfish (*Asterina pectinifera*) oocyte in relation to the surface actin remodeling. Before the onset of the bulge formation, the tension at the animal pole globally decreased, and started to increase after the onset of the bulge formation. Locally, at the onset of the bulge formation, tension at the top of the animal pole began to decrease, whereas that at the base of the bulge remarkably increased. As the bulge grew, the tension at the base of the bulge additionally increased. Such a change in the tension at the surface was similar to the changing pattern of actin distribution. Therefore, meiotic cell division was initiated by the bulging of the cortex, which had been weakened by actin reduction, and was followed by contraction at the base of the bulge, which had been reinforced by actin accumulation. The force generation system is assumed to allow the meiotic apparatus to move just under the membrane in the small polar body. Furthermore, a detailed comparison of the tension at the surface and the cortical actin distribution indicated another sophisticated feature, namely that the contraction at the base of the bulge was more vigorous than was presumed based on the actin distribution. These features of the force generation system will ensure the precise chromosome segregation necessary to produce a normal ovum with high accuracy in the meiotic maturation.

## Introduction

In meiotic maturation, the oocyte reduces the number of chromosomes to half through two successive meiotic cell divisions, in which the oocyte produces a normal ovum with high accuracy. Oocyte meiotic divisions are characterized by highly asymmetric division that occurs through dynamic surface actin remodeling [1], [2], [3], [4], [5], [6]. Morphological changes of the oocyte are inherently mechanical and arise from the spatially inhomogeneous tension at the surface [7], [8]. In addition, it has been shown that mechanical factors, including tensions at the surface, affect surface actin remodeling to dictate dramatic shape changes [9]. Thus, to fully understand the sophisticated spatiotemporal regulation of accurate oocyte meiotic divisions, it is important to focus on the spatiotemporal changes in the tension at the surface in relation to the surface actin remodeling.

In the starfish oocyte meiotic divisions, global and local actin remodeling has been observed [10]. Globally, actin filaments first accumulate near the vegetal pole and then near the animal pole before the bulge of the polar body extrusion. Locally, actin filaments decrease at the top of the animal pole and increase at the base of the bulge before the onset of the bulge formation. In the mouse oocyte [11], [12], meiosis progression is shown to be accompanied by global remodeling of surface architecture. Therefore, in spatiotemporal regulation of oocyte meiotic divisions, actin remodeling on various spatial scales from a tiny polar body scale to a larger whole oocyte scale is important. Further studies on the tensions at the surface on various spatial scales in relation to the actin remodeling are expected to unveil the mechanism of accurate oocyte meiotic divisions.

Studies from mechanical perspectives on the morphology and tension of meiotic cell division have been performed on the starfish oocyte using image-based quantification [13], [14], or by applying an external force with a glass plate. In these studies, the spatial resolution of the tension at the surface has been limited and provided little information near a polar body. Concerning symmetric cell division, the temporal change in tensions over the entire surface of the sea urchin egg have been determined by fitting *in vivo* cell shapes to a mathematical model [15]. A recent study by Koyama et al. has [16] adopted a similar strategy to estimate surface bending stiffness with another mathematical model, and successfully determined the stiffness over a whole cell cortex with high spatiotemporal resolution during symmetric cell division. The strategy of fitting *in vivo* cell shapes to a mathematical model is a powerful way to focus on the mechanical properties of a cell with high spatiotemporal resolution. One major consideration when applying the strategy to oocyte cell division, in which the changes in tensions on various spatial scales are all critical, is to acquire images having high spatial resolution and field of view [17] that are sufficient to detect the changes from a tiny polar body scale to a larger whole oocyte scale.

In this study, we first clarified the tensions at the surface in detail during meiotic cell division, which is highly asymmetric, by fitting *in vivo* cell shapes to a mathematical model. We adopted the Laplace formula that is a mathematical expression of the balance of forces of a thin elastic shell [18], of which the validity has long been carefully discussed in studies for equal cell divisions in sea urchin eggs [15], [19], [20], [21]. Starfish oocytes as an *in vivo* model have the advantage of simplifying the computation because of their spheroidal geometry. In order to detect the spatial change from a larger whole oocyte scale to a tiny polar body scale, rough and fine estimations of tensions at the surface were used in combination. We analyzed the images of oocytes during the meiotic cell division with an original computer program in which we approximated the surface shape data polynomially by means of a spline function [22] and calculated the changes in the tension at the surface of the oocyte in detail. Subsequently, these changes in tension were compared with the distribution of actin filaments. The analysis successfully clarified the characteristic features of the force generation system driving the morphological change of the oocyte which realizes meiotic cell divisions to produce a normal ovum with high accuracy.

## Materials and Methods

### Biological Materials

Individuals of the starfish, Asterina pectinifera, were collected from the Miura Peninsula, the Boso Peninsula, and the coast of Asamushi, Japan (authorized respectively by Misaki Marine Biological Station, The University of Tokyo; Marine and Coastal Research Center, Ochanomizu University; Research Center for Marine Biology, Asamushi, Tohoku University), and used as described previously [23]. Starfish oocytes were briefly cultured to remove follicle cells in Ca-free sea water (Ca-free Jamarin-U, Jamarin Lab., Oosaka, Japan) at 20°C. They were treated for 20 min at 20°C with 1 µM of 1-methyladenine (1-MeAde, Sigma-Aldrich, St. Louis, MO, USA) in order to make them resume meiosis. Maturing oocytes were inseminated 30 min after addition of 1-MeAde to elevate the fertilization envelope and to facilitate the observation of meiotic cell division.

### Image Acquisition and Calculation of Tension at the Surface – Rough Estimation of the Tension at the Surface of Whole Oocytes

**Figure 1:**
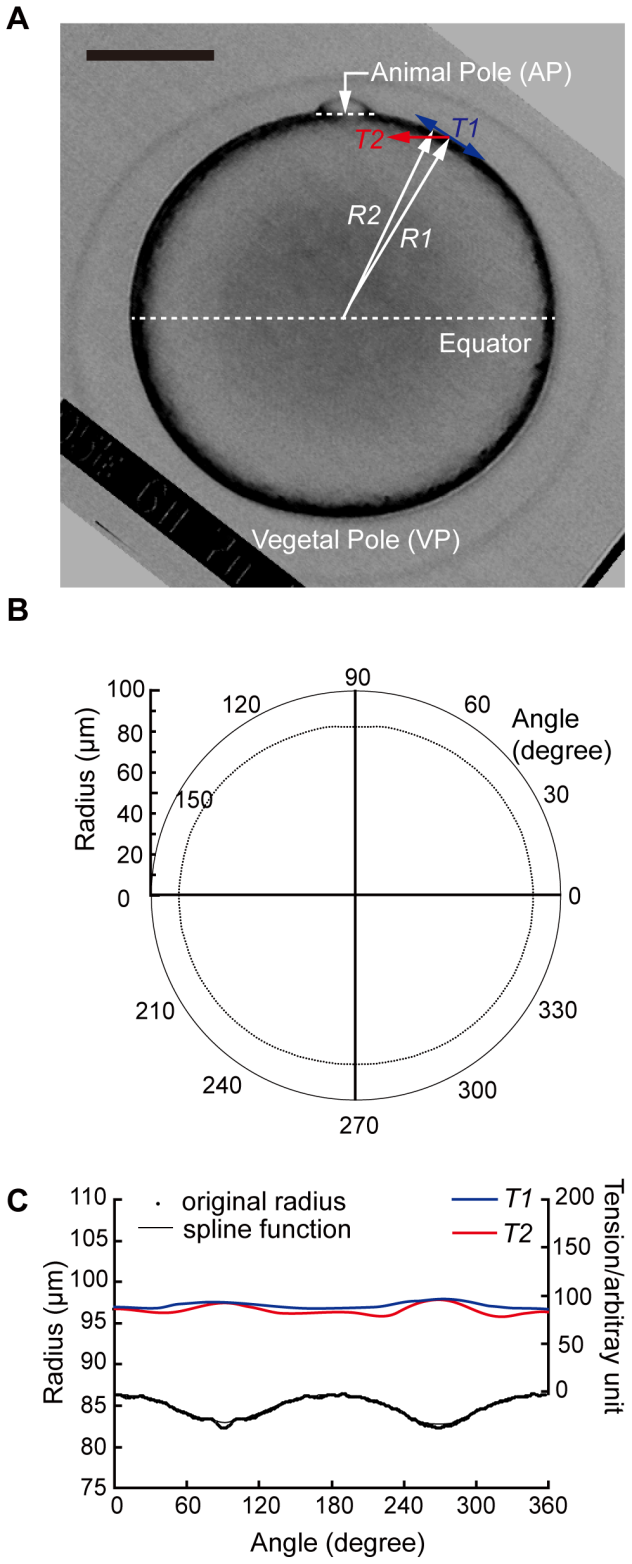
Procedure of rough estimation of the tension at the surface of whole oocytes. (A) Image of an oocyte at low resolution. Tension at the surface in the longitudinal direction (the direction joining pole to pole), *T1*, that in the latitudinal direction (the direction at right angles to the former), *T2*, and principal radii of curvature, *R1* and *R2* are indicated. Scale bar, 50 µm. (C) Binary image of the oocyte in (A) processed by NIH-Image, and the outline curve of the oocyte expressed in polar coordinates. (D) The radius of the oocyte and the tensions at the surface. The spline function accurately fits the radius of the original outline curve except for the animal pole.

The process of the rough estimation is shown in Fig. 1. The living oocyte was observed at low resolution with a photomicroscope (Optiphot, Nikon, Tokyo, Japan). Images of it were taken with a CCD camera (C2400-751, Hamamatsu Photonics, Hamamatsu, Japan) using a computer through an image processor (Argus 20, Hamamatsu Photonics, Hamamatsu, Japan). The image size was 480 pixels×480 pixels, and 1 pixel corresponded to 0.51 µm (Fig. 1A).

The principal radii of the oocyte surface curvature were calculated from the micrographs on the assumption that the oocyte was an ellipsoid of revolution whose axis running from the animal pole to the vegetal pole. In this rough estimation, as shown in Fig. 1B, the bulge of the polar body was removed, and the cell surface was outlined by the original macro program of the public domain NIH Image program (developed at the U.S. National Institutes of Health and available on the Internet at http://rsb.info.nih.gov/nih-image/).

From the cell image, the center of gravity was also obtained, then, the outline curve was expressed in polar coordinates (Fig. 1C) and the data were averaged so that the surface outline became symmetric along the animal-vegetal axis. With an original program on Mathematica (Wolfram Research Inc., Champaign, IL, USA), as shown in Fig. 1D, the surface outline was fitted to a four-dimension B-spline function, and the principal radii of curvature of the cell surface were obtained from the spline function formula. Then, the tension at the cell surface was calculated by the following formula [18], [19]:
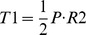


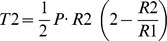
where *T1* is the tension at the surface in the longitudinal direction, *T2* is that in the latitudinal direction, and *P* is internal pressure, assumed to be 1. *R1* and *R2* are the principal radii of curvature of the cell surface (Fig. 1A). The rough estimation was done for four different oocytes.

### Image Acquisition and Calculation of Tension at the Surface – Fine Estimation of the Tension at the Surface of the Animal Hemisphere

**Figure 2:**
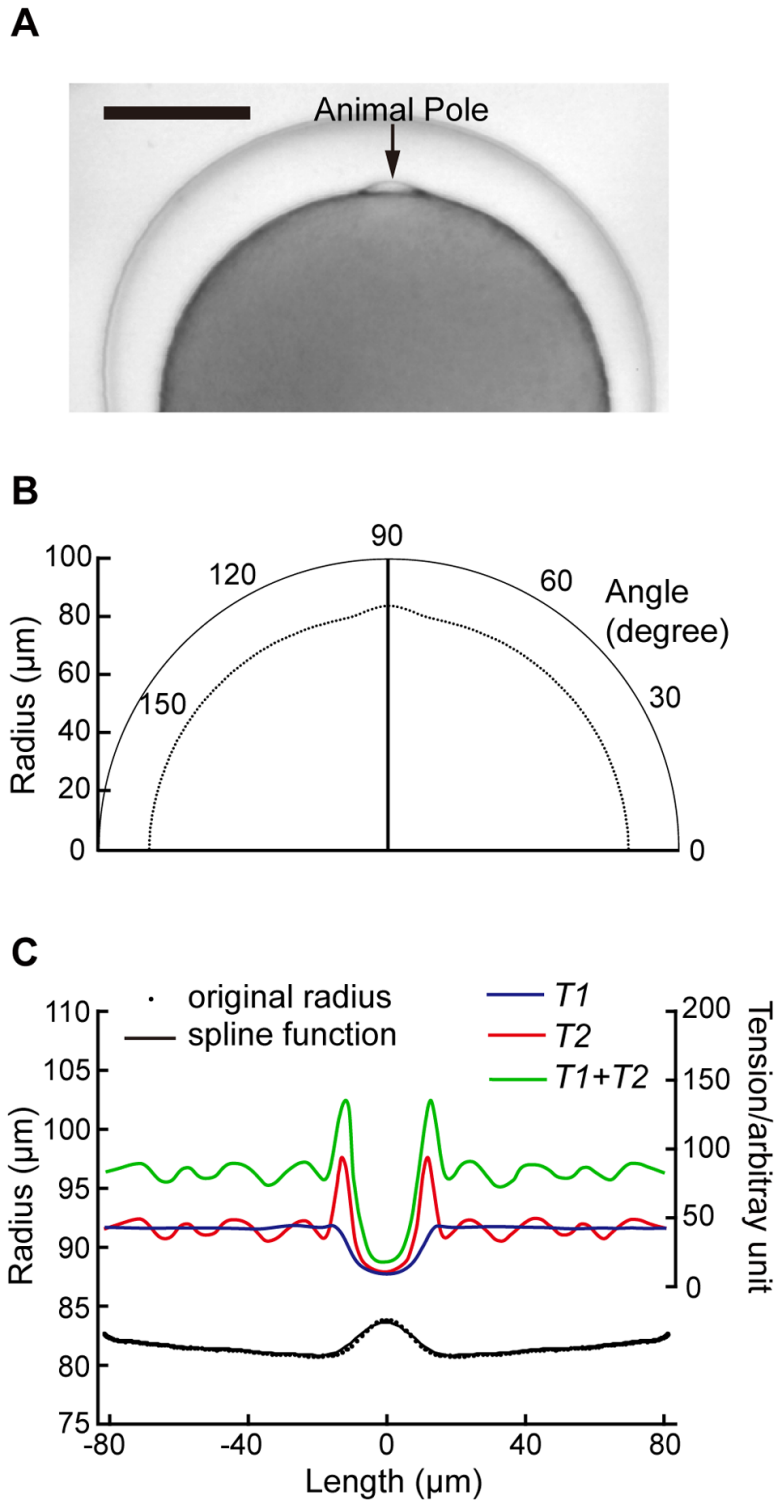
Procedure of fine estimation of tension at the surface of the animal hemisphere. (A) Image of the animal hemisphere of an oocyte at high resolution. Scale bar, 50 µm. (B) The outline curve in the animal hemisphere expressed in polar coordinates. (C) Tensions at the surface in the animal hemisphere and the radius of the oocyte versus the distance along the cord, vertical to the animal vegetal axis. The spline function accurately fits the radius of the original outline curve.

The process of fine estimation is shown in Fig. 2. The living oocyte was observed at high resolution with a photomicroscope (Optiphot-2, Nikon, Tokyo, Japan), and images of it were taken with a CCD camera (MicroImager, Xillix Technologies Corp., Richmond, Canada) by using a computer connected to an image processor (IP Lab, BioVision Technologies Inc., Exton, PA, USA). The image size was 1020 pixels×540 pixels and 1 pixel in the images corresponded to 0.22 µm (Fig. 2A). The cell surface including the polar body was outlined, and the outline curve was expressed in polar coordinates (Fig. 2B). The data were averaged to be symmetric along the animal-vegetal axis, then the tension of the animal hemisphere was calculated (Fig. 2C).

### Pseudocolor Images Near the Animal Pole

The pseudocolor images near the animal pole were illustrated based on the tension obtained with fine estimation. The relative tension, *T_r_*, was defined as:
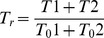
where *T_0_1* (the longitudinal direction) and *T_0_2* (the latitudinal direction) are the tensions at the surface with a vertical distance of 20 µm from the animal pole before bulging, or base of the bulge after the bulging.

Such a tension was represented with a pseudocolor on the three-dimensional (3D) cell surface whose image was restricted to the surface near the animal pole at the height of 20 µm from the animal pole, and the bulge of the polar body after the bulging. The fine estimation was done for four different oocytes.

## Results

### Changes in Tension of the Whole Oocyte

**Figure 3:**
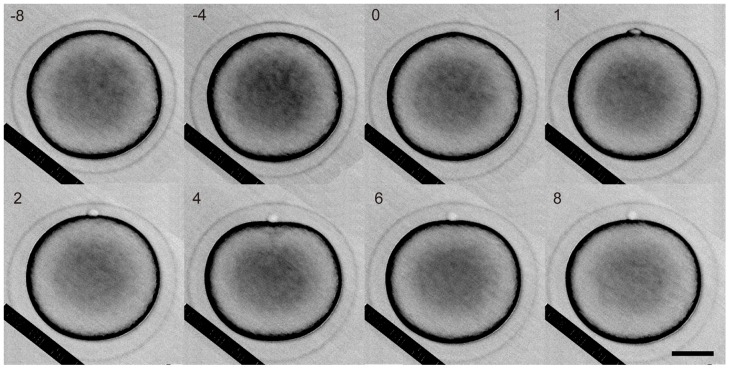
Shape change of a starfish oocyte during meiotic cell division. The top side is the animal pole. Numbers shown are the time after the onset of the bulge formation. Scale bar, 50 µm.

Spherical oocytes were selected for the analysis immediately after the 1-MeAde treatment. As shown in Fig. 3 and Video S1, during meiotic cell division, 8 minutes before the onset of bulging at the animal pole, the oocyte was slightly collapsed along the animal-vegetal axis. This event of collapse was reproducibly observed in three other oocytes.

The oocyte recovered to a spherical shape at the onset of the bulge formation (0 min in Fig. 3). It collapsed in the direction of the animal-vegetal axis again, 4 minutes after the onset of bulge formation. Then, during the polar body constriction, the oocyte recovered to a spherical shape (after 6 minutes in Fig. 3).

**Figure 4:**
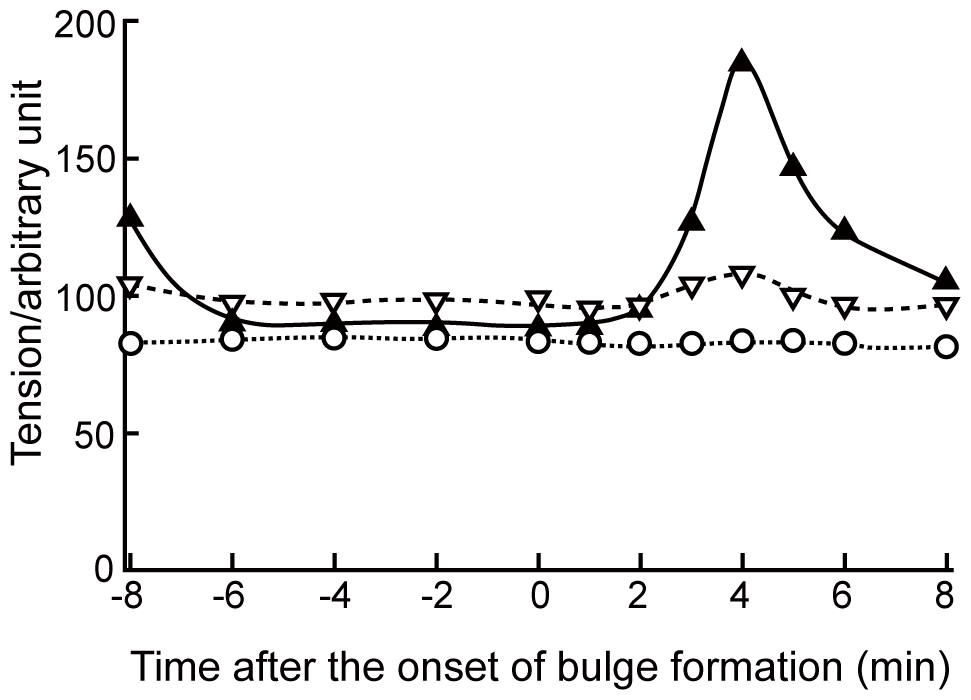
Global changes in the tension at the surface of the oocytes. The tension at the surface is calculated by the rough estimation based on the images shown in Fig.3. Abscissa: time after the onset of bulge formation. Ordinate: Tension, (*T1*+*T2*)/2, averaged over 20 degrees at the animal pole (solid line with filled triangle), that at the vegetal pole (broken line with open triangle), thdat at the equator (dotted line with open circle).

Following the method shown in Fig. 1, the morphological change of the oocyte during the meiotic cell division process was applied to the B-Spline function, and the tension was calculated and averaged over 20 degrees. In Fig. 4, we show the temporal changes in the averaged tension at the animal and vegetal poles, and the equator. The tension at the surface of the animal pole was greater than that of the vegetal pole 8 minutes before the bulge formation. Afterwards, the tension of the animal pole had become smaller than that of the vegetal pole from 6 minutes before the onset of the bulge formation to 1 minute after the onset of the bulge formation. In the analysis for three different oocytes, the tension at the animal pole was always smaller than that at the vegetal pole at this stage. Subsequently, of the animal pole, the tension increased up to twice, 4 minutes after the onset of the bulge formation. During the meiotic cell division, the tension was almost constant at the equator.

### Changes in the Tension around the Animal Pole in the Oocyte

**Figure 5:**
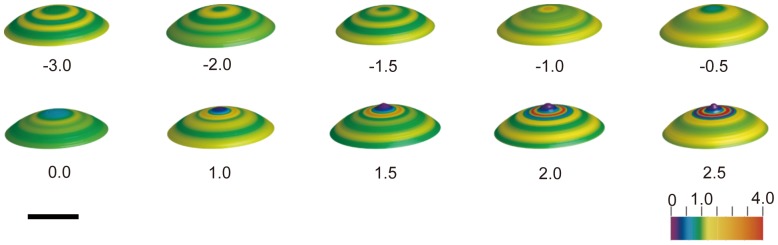
Time-course of relative tension around the animal pole. Relative tension, *T_r_*, at the surface, calculated by the fine estimation at a vertical distance of 20 µm below the animal pole and the bulge is represented with a pseudocolor on the 3D oocyte surface. The numbers shown below are the elapsed time (min) after the onset of the bulge formation. A color scale from 0 to 4.0 is shown at the bottom right of the figure. Scale bar, 50 µm.

To analyze the tension around the animal pole in detail, following the method shown in Fig. 2, we calculated the tension by using high resolution images. In Fig. 5, the changes in the relative tension, *T_r_*, around the animal pole are shown. The animal pole of the image showed green 3 minutes before the onset of bulging, which meant that *T_r_* was about 1. One minute before the onset of bulging, the narrow region, which was supposed to become a polar body, changed to yellow, indicating that *T_r_* was more than 1. Then, *T_r_* at the animal pole decreased to less than 1, just before the bulge formation. One minute after the bulge formation, *T_r_* started to increase at the position where the furrow was supposed to form. As the bulging grew, *T_r_* at the top of the polar body in the animal pole decreased further, and *T_r_* at the furrow increased to more than 3 as shown by a change to red 2.5 minutes after the onset of bulging.

**Figure 6:**
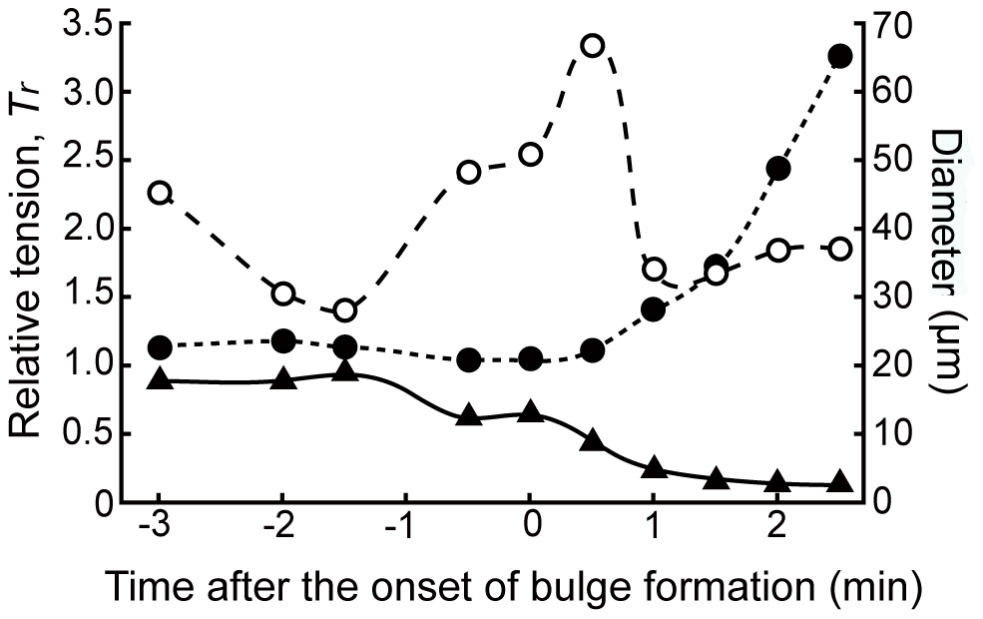
Characteristics of spatiotemporal changes in the tension at the surface around the animal pole. Time-course of the relative tension, *T_r_*, at the top of the animal pole (solid line with triangle), the maximum of *T_r_* at the base of the bulge (dotted line with filled circle), and the diameter of the maximum *T_r_* ring at the base of the bulge (broken line with open circle). Abscissa: time after the onset of bulge formation. Left ordinate: relative tension, *T_r_*. Right ordinate: distance of two peaks at the base of the bulge.

At *t* = −3.0 in Fig. 5, the maximum tension is circularly distributed approximately 20 µm away from the top of the animal pole. The temporal changes in *T_r_* at the top of the animal pole and those in the maximum *T_r_* are assumed to be critical processes for oocyte in determining the site of the bulge before the onset of the polar body formation, and in the growth of the bulge after the onset. Thus, the temporal changes in the maximum *T_r_*, those in *T_r_* at the top of the animal pole, and the diameter of the ring with the maximum *T_r_* were estimated and shown in Fig. 6. The relative tension, *T_r_*, at the top of the animal pole started to decrease before the onset of the bulge formation. One minute after the onset of the bulge formation, *T_r_* at the top of the animal pole was almost 0. In contrast to the *T_r_* at the animal pole, the maximum *T_r_*, as shown in Fig. 6, increased with the progression of the bulge formation. As seen in Fig. 6, the diameter of the ring with the maximum *T_r_* increased after the bulge formation. The diameter decreased until 1.5 minutes before the onset of the bulge formation. Afterwards, the diameter increased until 0.5 minutes after the onset of the bulge formation. At this time, the position of the dividing furrow is assumed to be fixed. Then, the diameter decreased, indicating that the dividing furrow had started to ingress.

## Discussion

In this study, we first analyzed the spatiotemporal change in the tension at the surface during meiotic cell division. Our method successfully clarified the local change in the tension at the surface of a tiny polar body, simultaneously with the global change over the whole cell surface. In previous studies, spatial resolution of the tension at the surface has been limited and provided little information about the tension at the surface in the polar body [13], [14], [24].

To simultaneously obtain data on global deformation of the whole oocyte and that around the animal pole with a small bulge of the polar body, we analyzed these with coarse and fine estimation procedures respectively. In the coarse estimation procedure, the shape changes of the animal pole were ignored. On the other hand, in the fine estimation procedure, the tension around the animal pole with a small bulge of the polar body was obtained successfully and with high accuracy. Another feature of our analysis is to use the spline function to present a complex cell surface outline during the meiotic cell division by approximating the outline as a smooth curve. In this research, a fourth-dimensional B-spline function in which the second differential coefficient was continuous [22] enabled us to approximate the cell surface with high accuracy.

On a global whole oocyte scale, as seen in Fig. 4, the tension around the animal pole was smaller than that around the vegetal pole from 6 minutes before the onset of the bulge formation to 1 minute after. Then, the tension around the animal pole started to increase relative to that in the vegetal pole. Similar polarization of the tension seems to be general across species based on the observation that mouse oocytes cortical tension is differing ∼2.5-fold between the cortex over the meiotic spindle and the opposite cortex [11]. The changes in the tension shown in our study before and during polar body formation were closely correlated with the globally unequal distribution of the actin filaments that has been observed by Hamaguchi et al. [10]. Until 1 minute after the onset of the bulge formation, accumulation of actin filaments around the vegetal pole has been observed, which correlates with greater tension around the vegetal pole than around the animal pole. One minute after the onset of the bulge formation, it has been observed that actin filaments started to accumulate around the animal pole, which correlates with increased tension around the animal pole compared to around the vegetal pole.

**Figure 7:**
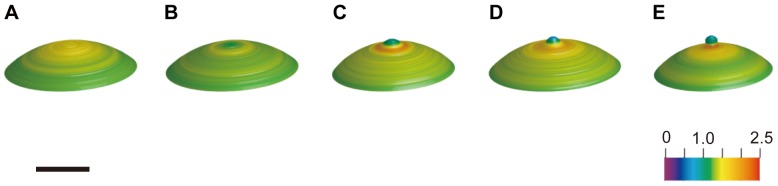
Time-course of actin distribution around the animal pole. Actin distribution (A) before and (B) at the onset of, and (C, D, E) after the bulge formation. Actin fluorescence distribution that had been analyzed previously [10] is shown in pseudocolor on the 3D cell surface whose image is restricted to the surface near the animal pole, at the height of 20 µm except for the bulge. For comparison with the data of the relative tension at the surface in Fig. 5, the fluorescence distribution is normalized by the ratio of actin fluorescent intensity around the animal pole to that at the surface at a vertical distance of 20 µm below the animal pole. A color scale indicating the normalized actin fluorescence from 0 to 2.5 is shown at the bottom right of the figure. Scal bar, 50 µm.

On a local tiny polar body scale, before the onset of the bulge formation, the actin filaments around the animal pole are reorganized to form the bulge of the polar body. In Fig. 7, the actin distribution related to the bulge formation is shown in 3D pseudocolor images to compare them with the tension. During the meiotic cell division, the pattern of actin distribution was similar to that of the tension in the surface. Before the onset of the bulge formation, the actin increased near the animal pole (Fig. 7A) as well as the tension (Fig. 5, *t* = −1.0). As the bulge grew, the actin at the animal pole decreased (Fig. 7B– E). In contrast, it increased at the furrow just before bulge formation and during bulging (Fig. 7C–E). Similarly, the tension decreased at the animal pole and increased at the furrow (Fig. 5, from *t* = −0.5 to 2.5).

**Figure 8:**
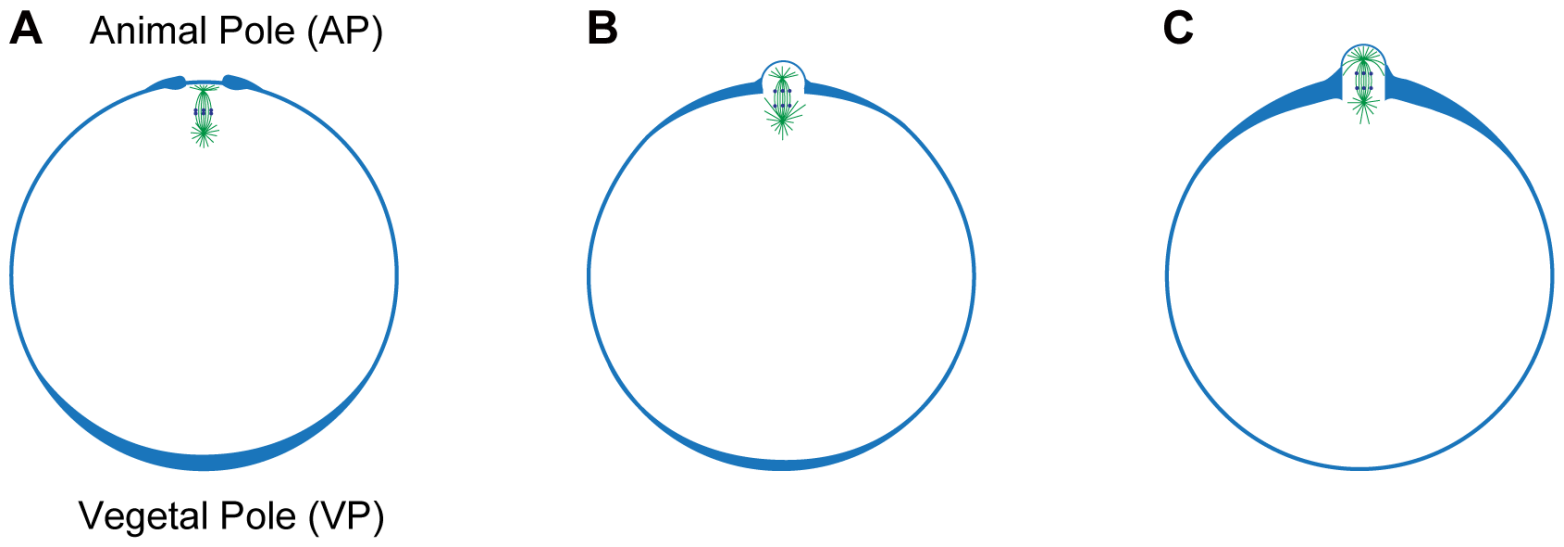
Force generation mechanism to ensure precise chromosome segregation for normal meiotic cell division. The magnitude of the tension is indicated by varying the thickness of the blue line. The meiotic apparatus is shown by the green line. Chromosomes are shown by the filled blue circles. (A) Before bulging started, the global tension around the animal pole is smaller than that in the vegetal pole. The meiotic apparatus exists just under the cell membrane at the animal pole. (B) After the onset of the bulge formation, the global tension around the animal pole starts to increase. Locally, the surface at the top of the animal pole weakens to form a bulge of the polar body; in contrast, the tension at the base of the bulge increases. The dividing furrow is formed in the equatorial plane of the meiotic apparatus. (C) As the bulge grows, the tension additionally increases at the base of the bulge. The meiotic apparatus moves into the bulge and is kept under the membrane. The position of the dividing furrow is in the equatorial plane of the mitotic apparatus.

By quantifying the global and local changes in the tension at the surface and by comparing the results with the distribution of actin filaments, as summarized in Fig. 8, we demonstrated the mechanism realizing morphological change of the oocyte during meiotic cell division. The polar body is assumed to form accompanied by movement of the cytoplasm [13]. Before the onset of bulge formation, as shown in Fig. 8A, the cell surface at the vegetal pole contracts due to the actin filament accumulation there, and the cytoplasm is pushed up to the animal pole. Next, the actin filaments start to accumulate in the animal pole, which induces the contraction there, and the polar body formation is assumed to be accompanied by the movement of the cytoplasm being pushed back to the vegetal pole. Afterwards, as illustrated in Fig. 8B, the tension at the animal pole decreases by actin reduction from the top of the animal pole. In contrast, the tension at the base of the bulge increases by actin accumulation at the base of the bulge. The actin accumulation at the bulge base creates a dividing furrow around the top of the animal pole. As the bulge grows, as shown in Fig. 8C, the tension at the furrow additionally increases as the actin accumulates. Finally, this furrow constricts the extruding bulge as a polar body.

A detailed comparison between the actin quantity and the tension at the furrow indicates the increased efficiency of maintenance of the tension at the furrow, which should help to ensure normal meiotic cell division in the force generation system, as shown in Fig. 8. The pattern of actin distribution was similar to that of the tension; however, at the furrow, the oocyte generated a maximum tension of 3.5, as shown in Fig. 6, which was larger than that simply presumed from the actin distribution. The relative tension at the furrow was 3.5, in contrast, the relative actin amount there is 2.0 in Hamaguchi et al. [10]. A possible mechanism to explain the result that the relative tension was larger than the actin amount can be hypothesized based on a simplified cortical model that relates the tension to the active stress as well as the elasticity of the actin network and the level of strain in that network. The active stress is supposed to have primary contribution for the tension because the cortical tension at the contractile ring in a dividing egg inhibited myosin ATPase activity by blebbistatin treatment dramatically decreased to be 10% less than that in the untreated egg [15]. Recent studies show that stretching of the actin cortex [25], [26] and actin filaments [27], which is closely correlated with the increase in the tension at the surface, enhances myosin II affinity to actin filaments. In addition, the efficiency of the force generation by the actin-myosin interaction is increased at the furrow with the parallel oriented actin filaments [6] relative to that in a randomly oriented actin meshwork even though the actin amounts are the same. The elastic modulus is probably secondary factor that contribute for the tension. The elastic modulus generally varies with actin concentration via some power law with a power of 1.4–1.8 [28], [29], thus the effect of the elasticity of a local strained cortex on the tension is assumed to increase according to the same power law.

Furthermore, the force generation system in Fig. 8 includes a mechanism to ensure precise chromosome segregation for normal meiotic cell division. In the mechanism, as shown in Fig. 8A, one pole of the mitotic apparatus at the metaphase of the first meiotic division is in contact with the animal pole surface [21]. Afterwards, as shown in Fig. 8B, the mitotic apparatus moves into the bulge with the movement of the cytoplasm from the animal pole to the vegetal pole. Then, a dividing furrow is formed in the equatorial plane of the mitotic apparatus (Fig. 8C), and the polar body includes a half spindle and the separated chromosomes. As illustrated in Fig. 8B–C, weakening the animal pole cortex during the polar body formation will achieve the movement of the mitotic apparatus kept under the cell membrane in the small polar body. This ensures precise chromosome segregation during the animal pole bulging.

## Conclusions

Our approach successfully clarified the spatiotemporal regulation of force generation that drives the morphological change of the oocyte during highly asymmetric meiotic cell division. The results indicate that the force generation system involves machinery to increase the efficiency of tension maintenance at the furrow, and to achieve the movement of the meiotic apparatus for precise chromosome separation, which will help to accurately produce a normal ovum in the meiotic cell division.

## Supporting Information

Video S1 **Shape change of a starfish oocyte during meiotic cell division.** The top side is the animal pole.
(AVI)

## References

[1] Longo FJ (1987) Actin-Plasma Membrane Associations in Mouse Eggs and Oocytes. Journal of Experimental Zoology 243: 299–309. 3655687 10.1002/jez.1402430215

[2] Pielak RM, Gaysinskaya VA, Cohen WD (2004) Formation and function of the polar body contractile ring in Spisula. Developmental Biology 269: 421–432. 15110710 10.1016/j.ydbio.2004.01.033

[3] Pielak RM, Hawkins C, Pyie A, Bautista J, Lee KG, et al (2005) Polar body formation in Spisula oocytes: Function of the peripheral aster. Biological Bulletin 209: 21–30. 16110091 10.2307/3593139

[4] Shimizu T (1981) Cortical Differentiation of the Animal Pole during Maturation Division in Fertilized-Eggs of Tubifex (Annelida, Oligochaeta).2. Polar Body Formation. Developmental Biology 85: 77–88. 7195850 10.1016/0012-1606(81)90237-2

[5] Shimizu T (1981) Cortical Differentiation of the Animal Pole during Maturation Division in Fertilized-Eggs of Tubifex (Annelida, Oligochaeta).1. Meiotic Apparatus Formation. Developmental Biology 85: 65–76. 7195849 10.1016/0012-1606(81)90236-0

[6] Shimizu T (1990) Polar Body Formation in Tubifex Eggs. Annals of the New York Academy of Sciences 582: 260–272. 2192600 10.1111/j.1749-6632.1990.tb21685.x

[7] Nakamura S, Hiramoto Y (1978) Mechanical-Properties of the Cell-Surface in Starfish Eggs. Development Growth & Differentiation 20: 317–327. 10.1111/j.1440-169X.1978.00317.x 37281468

[8] Freeman G, Ridgway EB (1988) The Role of Camp in Oocyte Maturation and the Role of the Germinal Vesicle Contents in Mediating Maturation and Subsequent Developmental Events in Hydrozoans. Rouxs Archives of Developmental Biology 197: 197–211. 10.1007/BF02439427 28305628

[9] Gowrishankar K, Ghosh S, Saha S, Rumamol C, Mayor S, et al (2012) Active Remodeling of Cortical Actin Regulates Spatiotemporal Organization of Cell Surface Molecules. Cell 149: 1353–1367. 22682254 10.1016/j.cell.2012.05.008

[10] Hamaguchi Y, Numata T, Satoh SK (2007) Quantitative analysis of cortical actin filaments during polar body formation in starfish oocytes. Cell Structure and Function 32: 29–40. 17575411 10.1247/csf.06034

[11] Larson SM, Lee HJ, Hung PH, Matthews LM, Robinson DN, et al (2010) Cortical Mechanics and Meiosis II Completion in Mammalian Oocytes Are Mediated by Myosin-II and Ezrin-Radixin-Moesin (ERM) Proteins. Molecular Biology of the Cell 21: 3182–3192. 20660156 10.1091/mbc.E10-01-0066 PMC2938384

[12] Azoury J, Lee KW, Georget V, Hikal P, Verlhac MH (2011) Symmetry breaking in mouse oocytes requires transient F-actin meshwork destabilization. Development 138: 2903–2908. 21653611 10.1242/dev.060269

[13] Hamaguchi MS, Hiramoto Y (1978) Protoplasmic Movement during Polar-Body Formation in Starfish Oocytes. Experimental Cell Research 112: 55–62. 564780 10.1016/0014-4827(78)90524-4

[14] Ikeda M, Nemoto SI, Yoneda M (1976) Periodic Changes in Content of Protein-Bound Sulfhydryl-Groups and Tension at Surface of Starfish Oocytes in Correlation with Meiotic Division Cycle. Development Growth & Differentiation 18: 221–225. 10.1111/j.1440-169X.1976.00221.x 37280850

[15] Miyoshi H, Satoh SK, Yamada E, Hamaguchi Y (2006) Temporal change in local forces and total force all over the surface of the sea urchin egg during cytokinesis. Cell Motility and the Cytoskeleton 63: 208–221. 16470543 10.1002/cm.20118

[16] Koyama H, Umeda T, Nakamura K, Higuchi T, Kimura A (2012) A High-Resolution Shape Fitting and Simulation Demonstrated Equatorial Cell Surface Softening during Cytokinesis and Its Promotive Role in Cytokinesis. Plos One 7. 10.1371/journal.pone.0031607 PMC3281004 22359606

[17] Miyoshi H, Adachi T (2012) Spatiotemporal coordinated hierarchical properties of cellular protrusion revealed by multiscale analysis. Integrative Biology 4: 875–888. 22689105 10.1039/c2ib20013a

[18] Timoshenko S, Woinowsky-Krieger S (1959) Theory of plates and shells. New York: McGraw-Hill.

[19] Hiramoto Y (1968) The Mechanics and Mechanism of Cleavage. In: PL M, editor. Aspects of Cell Motility. Cambridge: Cambridge University Press. 311–327.

[20] Hiramoto Y (1981) Mechanical properties of dividing cells. In: Zimmerman A, Forer A, editors. Mitosis/Cytokinesis. New York: Academic Press. 397–416.

[21] Rappaport R (1996) Cytokinesis in Animal Cells. Cambridge: Cambridge University Press.

[22] Ichida K, Yoshimoto F, Kiyono T (1976) Curve Fitting by a Piecewise Cubic Polynomial. Computing 16: 329–338.

[23] Satoh SK, Oka MT, Hamaguchi Y (1994) Asymmetry in the Mitotic Spindle Induced by the Attachment to the Cell-Surface during Maturation in the Starfish Oocyte. Development Growth & Differentiation 36: 557–565. 10.1111/j.1440-169X.1994.00557.x 37281517

[24] Hiramoto Y (1976) Mechanical-Properties of Sea-Urchin Eggs.3. Visco-Elasticity of Cell-Surface. Development Growth & Differentiation 18: 377–386. 10.1111/j.1440-169X.1976.00377.x 37281788

[25] Mohan K, Iglesias PA, Robinson DN (2012) Separation anxiety: Stress, tension and cytokinesis. Experimental Cell Research 318: 1428–1434. 22487096 10.1016/j.yexcr.2012.03.028 PMC3372636

[26] Effler JC, Iglesias PA, Robinson DN (2007) A mechanosensory system controls cell shape changes during mitosis. Cell Cycle 6: 30–35. 17245114 10.4161/cc.6.1.3674 PMC4638380

[27] Uyeda TQP, Iwadate Y, Umeki N, Nagasaki A, Yumura S (2011) Stretching Actin Filaments within Cells Enhances their Affinity for the Myosin II Motor Domain. Plos One 6. 10.1371/journal.pone.0026200 PMC3192770 22022566

[28] Hinner B, Tempel M, Sackmann E, Kroy K, Frey E (1998) Entanglement, elasticity, and viscous relaxation of actin solutions. Physical Review Letters 81: 2614–2617.

[29] Gardel ML, Valentine MT, Crocker JC, Bausch AR, Weitz DA (2003) Microrheology of entangled F-actin solutions. Physical Review Letters 91. 10.1103/PhysRevLett.91.158302 14611506

